# Distal fibular excision: A review of the literature and presentation of our reconstruction technique case series

**DOI:** 10.1016/j.ijscr.2021.01.105

**Published:** 2021-02-11

**Authors:** Ashley Lamb, Joseph Mueller, Ezra Levy, Janet L. Hobbs, Earl Brien

**Affiliations:** a Graduate Medical Education, Community Memorial Health System, Ventura, CA, USA; b Community Memorial Health System| Cedars-Sinai: Cedars-Sinai Health System

**Keywords:** Case series, Neoplastic lesions, Distal fibular excision

## Abstract

Distal fibula resection is a procedure that has been described as early as 1938 for the treatment of neoplastic lesions. There have been several techniques described for the reconstruction of the remaining tibiotalar joint to prevent deformity and maintain as much function as possible. While these reconstruction techniques provide an option for limb salvage with the removal of disease, patients are faced with chronic pain, loss of function, valgus instability, need for long term orthosis, early arthritis, or significant morbidity related to proximal dissection and disruption of native knee anatomy.

We present a case series that is compliant with PROCESS^1^ criteria to demonstrate the effective treatment with distal fibular excision and a reconstruction technique inspired by the original Tommy John procedure of the elbow. This procedure has allowed these two patients long-term stability of the ankle, maintenance of full function, and high levels of function. The first case is a 23-year-old female with high-grade osteosarcoma and the second patient is a 19-year-old female with Ewing sarcoma. Details of the procedure as well as clinical and radiographic follow up of these two patients will be described.

## Highlights

• Distal fibula resection is a procedure that has been described as early as 1938 for the treatment of neoplastic lesions.
• Medial instability of the elbow can be traced back to the literature as far as 1946.
• The described technique can preserve long-term tibiotalar congruity and stability, allowing these patients to return to near normal function.

## 1 Case reports

### 1.1 Case 1

**Fig. 1:**
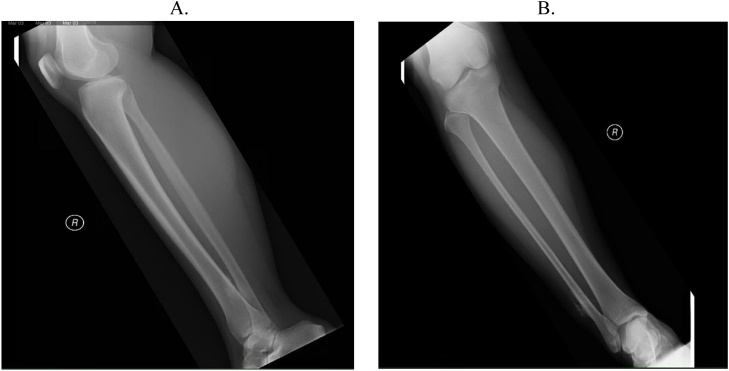
(A and B) Initial radiographs on presentation showing a soft tissue mass adjacent to the distal fibula vs. juxta cortical mass with punctate calcification centrally. Differential diagnosis radiographically included juxta cortical chondroma and parosteal osteosarcoma.

**Fig. 2:**
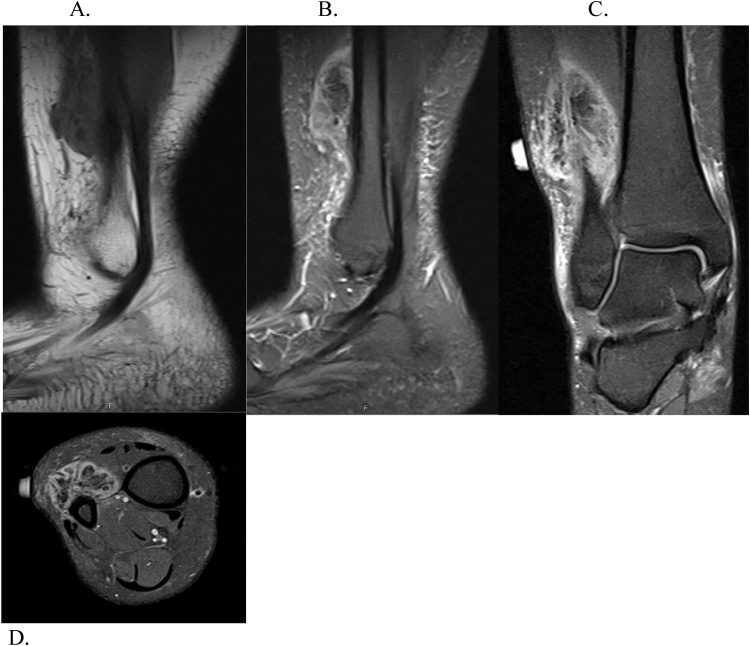
A) Sagittal T1 turbo spin echo sequence of high-grade osteosarcoma sound on preoperative imaging of Patient #1. B) Sagittal short T1 inversion recovery, turbo echo sequence. C) Coronal proton density, turbo spin echo, fat suppressed sequence.

**Fig. 3:**
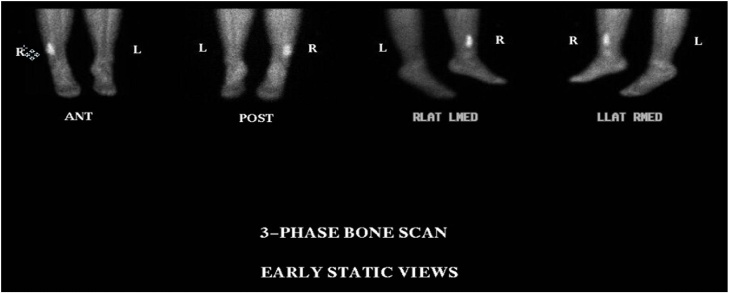
Bone scan of Patient #1 showing focal area of intense increased activity in the distal right fibula.

**Fig. 4:**
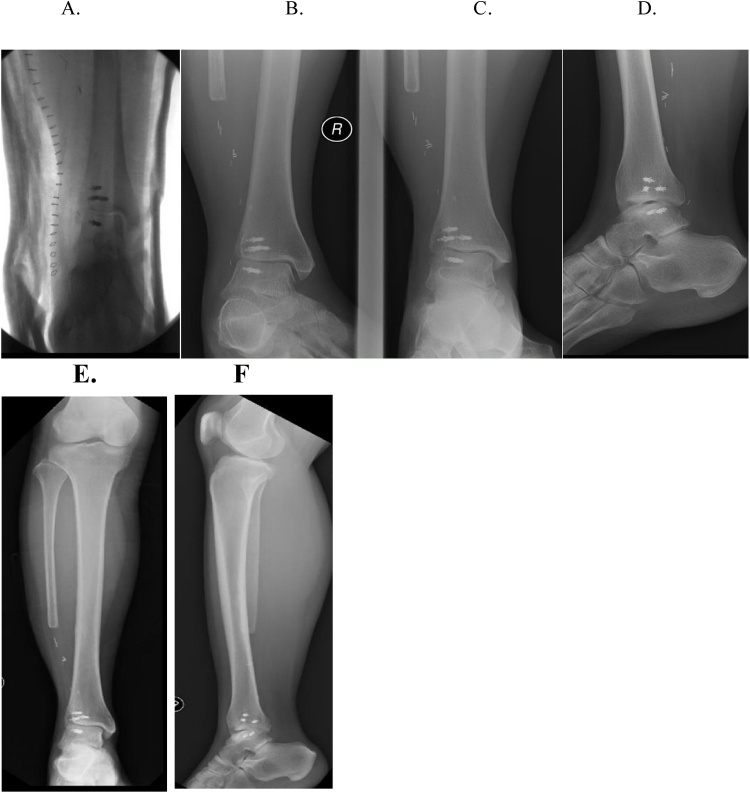
A) Immediate postoperative radiographs of patient #1 A, B, C, D) Two years post-op. E, F) Eight years post-op.

A 23-year-old Asian female with no prior medical history presented with swelling and pain to the right lateral ankle in early 2010. At the time of presentation, she already had developed paresthesia of the foot's dorsum consistent with peroneal nerve irritation. Radiographs, MRI, and bone scan demonstrated juxtacortical lesions of the right distal fibula (Fig. 1, Fig. 2, Fig. 3). She underwent an open biopsy, which was analyzed by an interdepartmental pathology review team. The pathology report was sent out for further evaluation to the Mayo Clinic in Rochester, Minnesota. Histologic evaluation unanimously yielded a diagnosis of high-grade osteosarcoma. She underwent neoadjuvant chemotherapy treatment with high-dose methotrexate, ifosfamide, Adriamycin, and Cisplatin with a cumulative Adriamycin dose of 135 mg/m2. After chemotherapy was completed, MRI scans were obtained. The patient then underwent uncomplicated en bloc resection of the right distal fibula with close margins. The distal tibia was partially excised. Neurolysis of the deep peroneal nerve was done with the sacrifice of the distal portion of the superficial peroneal nerve, and peroneal tendon transfer reconstruction of the lateral collateral ligament, talus to the tibia (Fig. 4). Operative pathology demonstrated high-grade osteosarcoma of the distal fibula's surface and metaphysis, with satellite nodules extending into the surrounding soft tissue and biopsy tract. Tumor necrosis was 90 %. Postoperatively, she was placed in a three-sided plaster short leg splint and maintained non-weight bearing status. Two weeks post-op, her staples were removed. She was transitioned to a CAM walker boot and continued with her non-weight bearing status until six weeks postoperatively when she was allowed to progressively transition to weight-bearing. She resumed adjuvant chemotherapy with high-dose methotrexate, ifosfamide, Adriamycin, and Cisplatin starting at one-month post-op for five months long treatment. Follow up local MRI and Chest CT scans have remained negative for local recurrence or distal metastasis.

By two years post-op, the patient had returned to full unrestricted activity, including high level organized dancing. At her most recent follow-up visit, now eight years post-op and age 32, she continued to have no functional limitations or pain. She works as a nurse and can complete 10 -h shifts without difficulty. She wears regularly available supportive athletic shoes. She reported decreased sensation over the dorsal lateral three toes consistent with her sacrificed superficial peroneal nerve at surgery. She had 5/5 strength in active inversion and eversion against resistance. Her great toe and ankle plantar flexion and dorsiflexion were actively intact with 5/5 strength. Active ankle dorsiflexion was10 degrees. The active plantar flexion of the ankle was 20 degrees.

### 1.2 Case 2

**Fig. 5:**
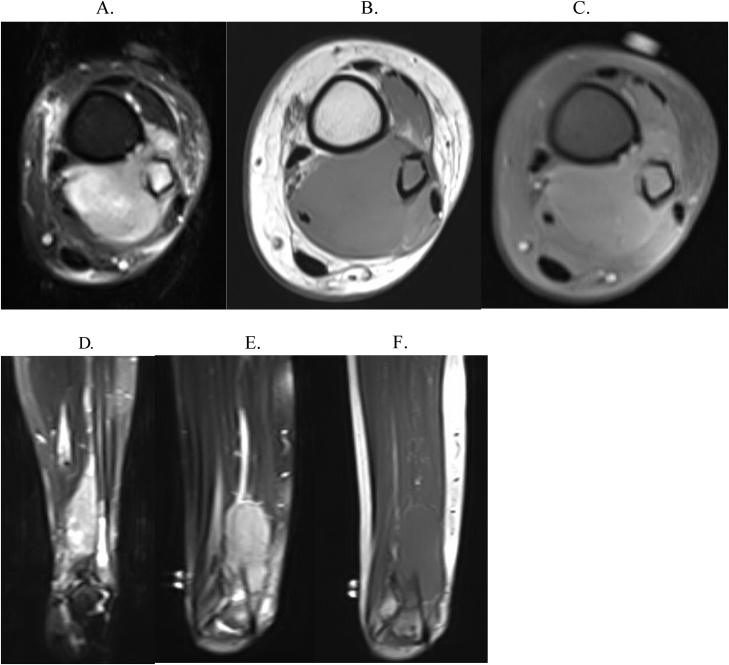
MRI of the left tibia/fibula of patient #2 A) Axial STIR sequence B) Axial TSE T1 C) Axial TSE T1 FS. D) Coronal STIR. E) Sagittal FSE T2 FS. F) Sagittal T1.

**Fig. 6:**
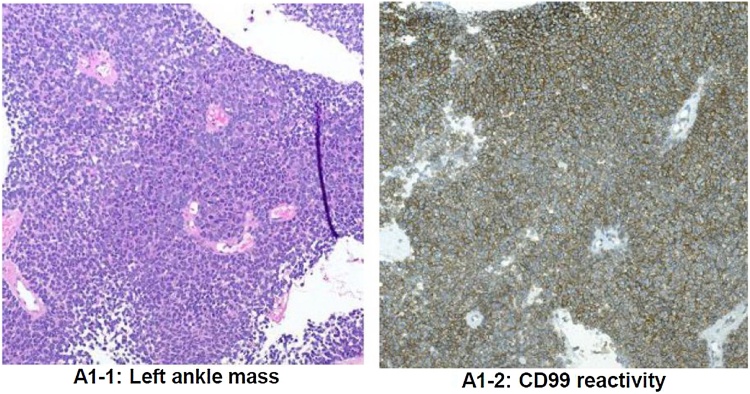
H&E and CD99 stains of CT biopsy tissue from the left distal fibula of Patient #2.

**Fig. 7:**
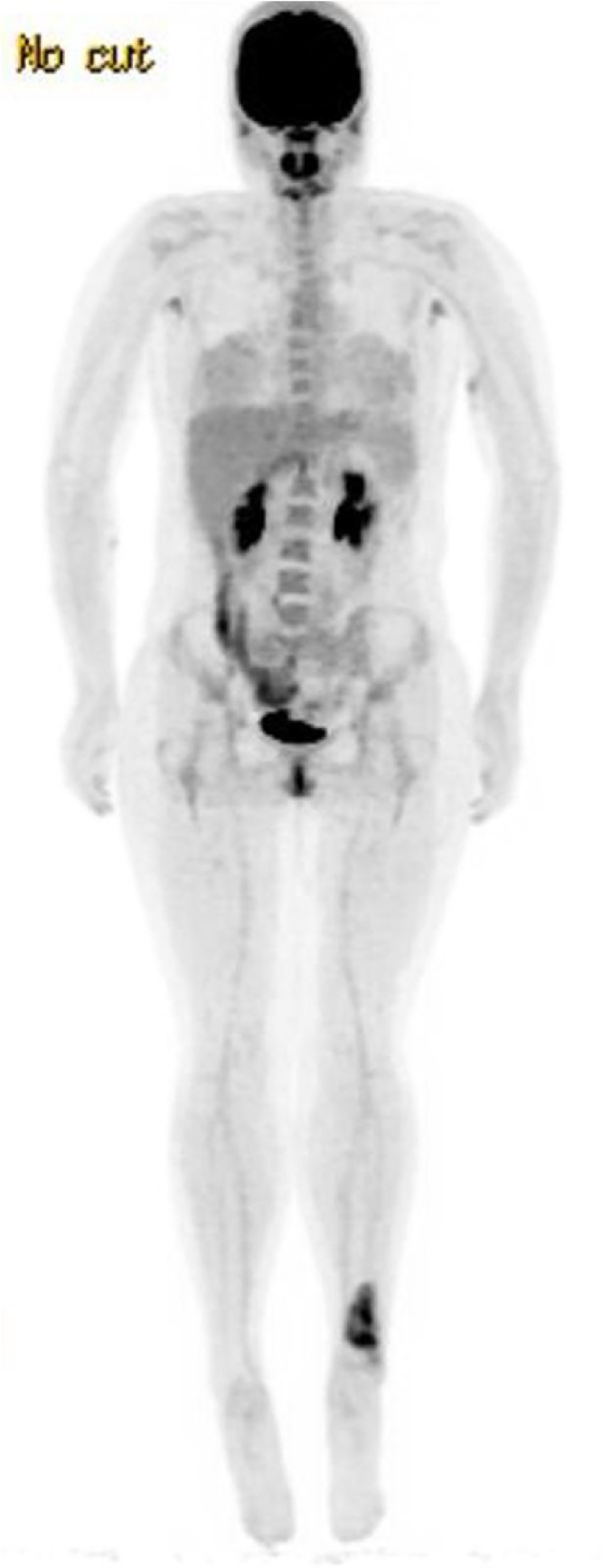
Whole body PET/CT scan of patient #2.

**Fig. 8:**
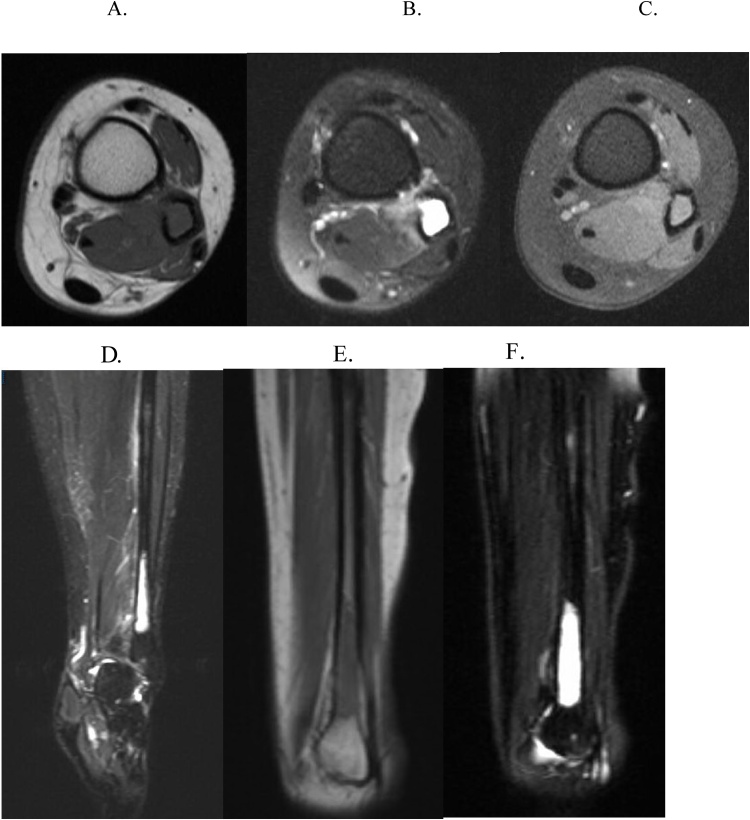
MRI of the left distal tibia/fibula in patient #2 after neoadjuvant chemo and radiation therapy. A) Axial T1. B) Axial T2 FS. C) Axial T1 FS. D) Coronal STIR. E) Sagittal T1. F) Sagittal T2 FS.

A 19-year-old Caucasian female with no past medical or surgical history. She presented to the orthopedic oncology clinic three months after a sudden onset of a traumatic left ankle swelling. An outside provider placed her in a CAM walking boot for a presumed ankle sprain; her swelling eventually subsided. After removing the boot and returning to unrestricted activity, the swelling returned. An MRI was done, which showed a large distal fibular mass with characteristics consistent for Ewing Sarcoma (Fig. 5). She underwent a CT guided biopsy on the same day of the initial consultation, which demonstrated a small round blue cell lesion that was CD99 positive, FLI 1 positive, and FISH positive for EWSR 1, confirming the diagnosis of Ewing Sarcoma (Fig. 6). The Hematology/Oncology service promptly saw her, and staging studies were negative for distant metastasis (Fig. 7). Pre-chemotherapy echocardiogram demonstrated an ejection fraction of 62 %. She was started on a neoadjuvant regimen of vincristine, doxorubicin, and cyclophosphamide. She also underwent a pre-operative radiation therapy regimen at a total dose of 4500 cGy broken up into 25 fractions. After completing her neoadjuvant chemotherapy and radiation treatment, a repeat MRI scan showed a significant decrease in the distal fibular lesion and soft tissue component (Fig. 8). She continued to be followed with surveillance MRI scans for local recurrence until a decision was made between the patient, family, and treating specialties to proceed with surgical treatment.

**Fig. 9:**
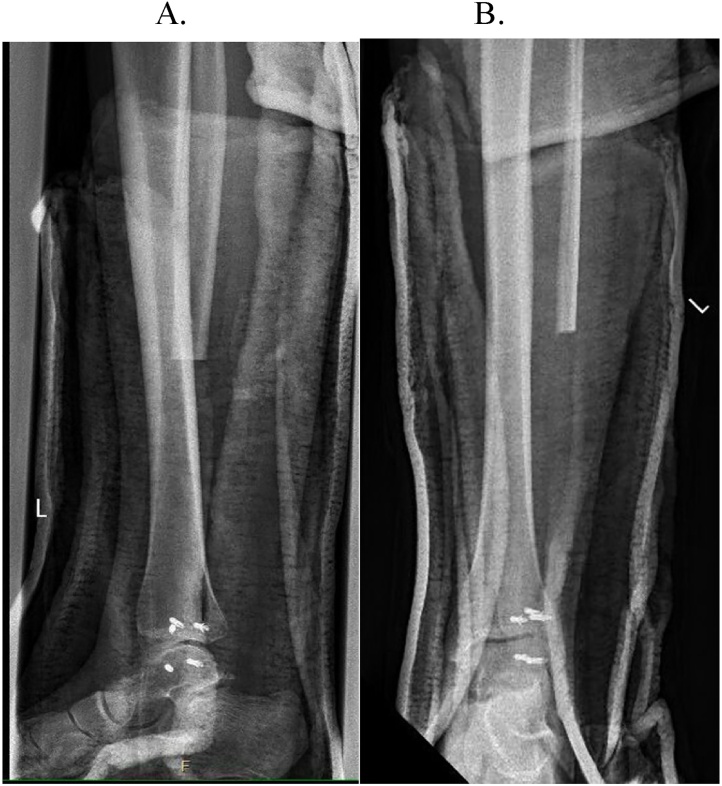
A and B) Immediate postoperative radiographs of patient #2.

Just over one year from the initial presentation, she underwent en bloc resection of the distal left fibula, neurolysis of branches of both the superficial peroneal and deep peroneal nerves, local flaps, and reconstruction of the lateral ankle ligaments using the peroneus brevis (Fig. 9). She was immobilized in the immediate postoperative period with a three-sided short leg splint and was instructed to be non-weight bearing.

She did very well ten months postoperative without evidence of recurrence and intact mortise on imaging studies (MR/CT/XR). She was ambulating in a high-top shoe without discomfort or major gait disturbance. Sensation was intact distally in the foot in the sural, saphenous, superficial peroneal, deep peroneal, medial, and lateral plantar nerve distributions. Gross motor was intact in the tibialis anterior, gastrocnemius, extensor hallucis longus, and flexor hallucis longus. She was encouraged to continue supportive shoe ware and limit ambulation on uneven surfaces until 15 months post-op. She will have continued to follow up with serial imaging.

## 2 Operative technique

Both patients underwent the procedure in supine position, under general anesthesia, and they had non-sterile tourniquets. Standard sterile technique were used to prepare and drape the lower extremities. Lateral based incisions were made, ascertaining to ellipse out any prior incisions or biopsy tracts. Fasciocutaneous flaps were made broadly to the anterior musculature. The musculature of the tibialis anterior were dissected, which were partially left with the tumor bed. The ligaments were removed distally. Proximally, the dissection were also carried around the fibula, leaving approximately a 4 cm margin above the tumor site, and the fibula were osteotomized.

**Fig. 10:**
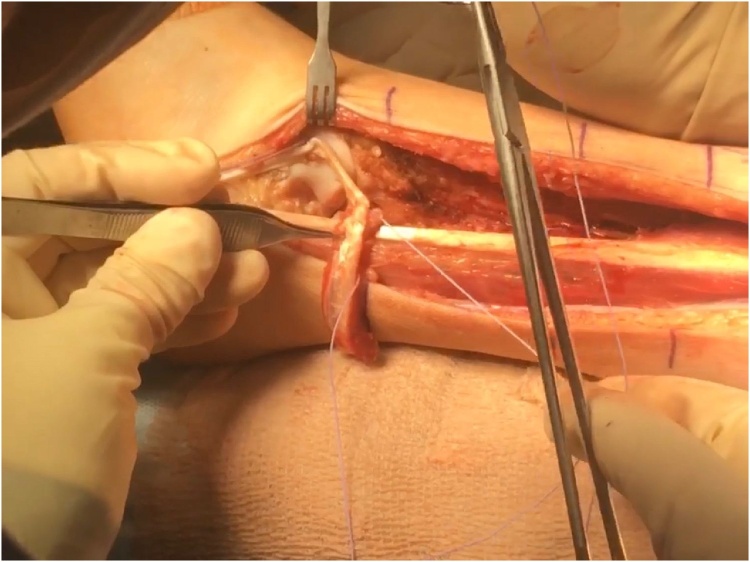
Dissection of the peroneus brevis muscle for lateral ankle ligament reconstruction.

**Fig. 11:**
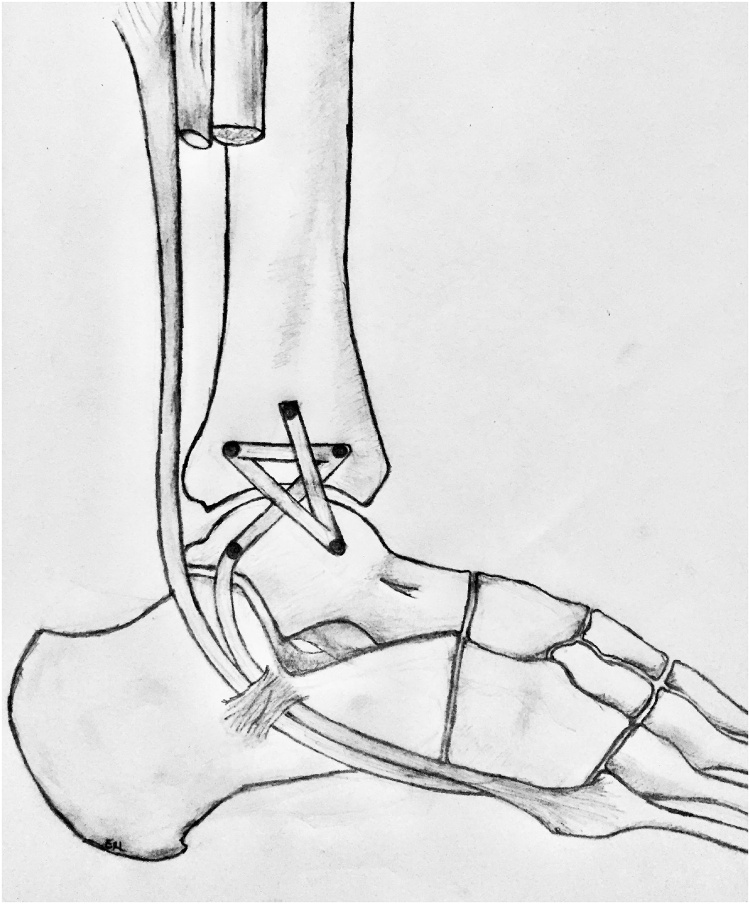
Peroneus brevis tendon suture anchor reconstruction of lateral ankle ligaments after distal fibula excision.

Posteriorly, small muscles were left with the fibula, and the interosseous membrane were incised. An osteotome were used to remove the tibia with the fibula to achieve maximum margins. The dissections were carried distal to proximal as the flaps were elevated. The blood vessels including the peroneal vessel, which feeds the fibula were ligated with clips and silk ties. Once these were completed, attention were directed to removing the fibula en bloc, with neurolysis of the deep and superficial peroneal nerve as indicated depending on the particular tumor. The peroneus brevis tendon were removed from its bed to reconstruct the lateral collateral ligaments (Fig. 10). The peroneal tendon were reconstructed with suture anchors in a stellate pattern (Fig. 11). All margins were confirmed to be clear, and the fasciocutaneous flaps were then approximated using 2−0 Vicryl, and subcutaneous tissues with 3−0 Vicryl.

## 3 Discussion

While medial instability of the elbow can be traced back to the literature as far as 1946, the reconstruction procedure for this valgus elbow instability was first described and credited to Drs. Jobe, Stark, and Lombardo in 1986 [9]. This concept of soft tissue reconstruction using a figure of eight configuration has motivated the development of the reconstruction technique used for the two cases described in this study.

Several other modalities have been described in the literature to manage pathologies requiring distal fibular excision, and several others have described the complications encountered in caring for these patients [[24], [25], [26], [27]]. Carrel reported the earliest case found during our literature review in 1938 [1].They described two patients, one with hemangioma of the fibula's distal portion and the other with Ewing's sarcoma. In these cases, the distal fibula were resected, and the proximal portion of the fibula were transplanted distally to maintain the ankles’ lateral stability. This procedure has been reported in several subsequent publications. In 1979 Persson and Rydholm described their experience in a case of chondrosarcoma. They reported normal patient motility with no instability at three years to follow up [12]. In 1986, Capanna described their results with this 180-degree transposition of the proximal fibula in two patients and several other techniques [2]. Their experience with proximal fibular transposition was in one patient with chondrosarcoma and one with Ewing Sarcoma. The chondrosarcoma patient had a two-year follow-up and reportedly had excellent functional results with no pain, full mobility, and no instability. The Ewing Sarcoma patient had a 0.5 year follow up at the time of publication and had no pain or instability but reduced mobility. In 1997, Herring described a case of an eight-year-old girl who had fallen from a moving vehicle [6]. She sustained significant tissue loss and loss of a 7.5 cm section of the distal fibula. This was eventually treated with proximal fibular transposition and reconstruction of the lateral ankle ligaments with the distal portion of the peroneus brevis tendon. The patient did well but unfortunately developed a hindfoot valgus deformity that required return to the operating room for a supramalleolar tibiofibular osteotomy at 16 years of age. They presented a 28 year follow up of the patient walking without an external device, had no pain, and had a uniform gait with the foot in a neutral position. She was able to toe walk, heel walk, and could heel-toe uniform gait without a limp. She could wear various heel heights without difficulty. The foot could be dorsiflexed actively to neutral, and there was 30° active plantar flexion. De Gauzy published a report of a 13-year-old with osteosarcoma who underwent this proximal fibular transposition with the modification of placing the obliquely to allow better congruency of the proximal fibular articular surface to the talus [4].

Several additional reconstruction techniques have been described. Capanna and colleagues described a case series review of 11 patients using five different reconstruction techniques [2]. Two of the patients were discussed in the previous paragraph. All of their patients had benign lesions and were treated with excision of the lesions and retention of the lateral malleolus with or without intertibiofibular arthrodesis. While all three of these patients had excellent functional results at a minimum one-year follow-up, this treatment modality is not an option in malignant neoplasm. This treatment modality has also been successfully carried out and described by Shoji et al. in 1970 for the treatment of aneurysmal bone cyst, and by Piocciona and Martorana for the treatment of chondromyxoid fibroma [3]. Capanna had an additional three benign lesions that were treated similarly but with the addition of curettage of the lateral malleolus and a cortical graft. Each of these patients achieved excellent functional outcome as well. They then describe two patients with Ewing sarcoma treated with complete fibular excision and stabilization using the peroneus brevis tendon that closely mirrored the technique used in the cases presented in this study. After resecting the distal fibula, Capanna's group sutured the distal ends of the peroneal tendons to the distal tibia's lateral side. They found these patients to have reduced joint mobility and stability.

Monson, Vojdani, Dean, and Louis-Ugbo did a more recent description of this procedure in 2014 [10]. In their technique, the peroneus brevis tendon was divided proximally at the myotendinous junction and secured to the lateral tibia and reflected in a manner that recreated the anterior tibiofibular and calcaneofibular ligaments. The respective ligamentous reconstructions were tensioned at 30 degrees plantarflexion and neutral plantarflexion, respectively, to match their anatomic biomechanical tensions. All three of their patients had excellent functional outcomes with no restrictions in activities of daily living.

## 4 Conclusion

Medial elbow instability reconstructive surgery, and the concept of soft tissue reconstruction using figure of eight configuration, has motivated the development of this novel lateral ankle reconstruction technique in patients requiring distal fibula resection. The described technique using the peroneus brevis to reconstruct the lateral ankle ligament complex can preserve long-term tibiotalar congruity and stability, allowing these patients to return to near normal function.

## Conflicts of interest

There are no conflicts of interest from any authors on this manuscript

## Funding

There are no funding sources on this manuscript.

## Ethical approval

The study is exempt from ethical approval.

## Consent

Written informed consent was obtained from the patient for publication of this case report and accompanying images. A copy of the written consent is available for review by the Editor-in-Chief of this journal on request.

## Author contributions

**Ashley Lamb, Joseph Mueller:** Conceptualization, visualization and design.

**Ezra Levy, Earl Brien:** Data curation and interpretation.

**Joseph Mueller, Ashley Lamb:** Interpretation and formal analysis, organization of figures.

**Ezra Levy, Ashley Lamb, Joseph Mueller, Earl Brien:** Methodology.

**Janet L Hobbs:** Writing - review & editing.

**Ezra Levy:** Project Administration and study supervision.

All authors read and approved the final manuscript.

## Registration of research studies

N/A.

## Guarantor

Ezra Levy, DO.

## Provenance and peer review

Not commissioned, externally peer-reviewed.
